# Modern Upper-Limb Rehabilitation Interventions in Stroke Patients with Spasticity

**DOI:** 10.3390/jcm15041560

**Published:** 2026-02-16

**Authors:** Ana Maria Bumbea, Rodica Trăistaru, Elena-Anca Târtea, Alexandra Oltea Dan, Adina Turcu-Stiolica, Daniela Matei, Simona Pătru, Bogdan Stefan Bumbea, Cristiana Octaviana Daia

**Affiliations:** 1 Faculty of Nursing, Medical Rehabilitation, University of Medicine and Pharmacy of Craiova, 200349 Craiova, Romania; anamariabumbea@yahoo.com (A.M.B.);; 2 Faculty of Medicine, Neurology, University of Medicine and Pharmacy of Craiova, 200349 Craiova, Romania; 3 Faculty of Medicine, Ophthalmology, University of Medicine and Pharmacy of Craiova, 200349 Craiova, Romania; 4 Faculty of Pharmacy, University of Medicine and Pharmacy of Craiova, 200349 Craiova, Romania; adina.turcu@gmail.com; 5 Maternity ICU Department, Vâlcea Emergency Hospital, 240284 Ramnicu Valcea, Romania; 6 Faculty of Medicine, University of Medicine and Pharmacy Carol Davila, 050474 Bucharest, Romania

**Keywords:** stroke, neuroplasticity, grip rehabilitation, spasticity, shock wave therapy, functional, electric stimulation

## Abstract

**Background:** Upper-limb rehabilitation is a decisive factor in improving the quality of life for patients who have experienced a stroke. Modern rehabilitation techniques promote the recovery of upper-limb functionality and prehension, contributing to a reduction in disability. **Materials and Methods:** This retrospective observational study aimed to highlight improvements in prehension through the application of current actual and modern rehabilitation techniques targeting key muscle groups involved in upper-limb recovery. Data from a total of 52 patients were identified and categorized into two groups based on the specific rehabilitation protocols they received during their hospitalization: a study group and a control group. Both groups underwent individualized rehabilitation, differing only in the type of electrotherapy applied: the study group received functional electrical stimulation (FES) and shock wave therapy (RSWT), while the control group received conventional electrical stimulation. **Results:** After adjusting for baseline differences in severity and time since stroke, patients in the study group demonstrated a significantly greater improvement in functional parameters compared to the control group. The results show us a significant improvement of functionality after RSWT and FES in the study group, with values from 0.28 ± 0.28 to 0.99 ± 0.36 (*p*-value < 0.001) regarding Hand Grip, suggesting that the treatment effect persists even when initial clinical advantages in the control group are accounted for. Muscle force increased from 0.39 ± 0.54 to 7.67 ± 3.89, *p*-value < 0.001. **Conclusions:** The combined application of functional electrical stimulation and shock wave therapy, as modern rehabilitation interventions, provided additional benefits in upper-limb and prehension rehabilitation compared to classical electrical stimulation alone. Our findings suggest that the combined application of RSWT and FES is strongly associated with improved upper-limb recovery, even after adjusting for baseline clinical imbalances. While these results support the integration of these modern techniques into stroke protocols, further prospective randomized controlled trials are needed to confirm the definitive treatment advantage over conventional methods.

## 1. Introduction

Stroke remains a major global health burden and a leading cause of long-term disability worldwide. According to the World Stroke Organization and World Health Organization reports, stroke is currently the second leading cause of death and the third leading cause of disability globally, with a substantial proportion of survivors experiencing persistent motor impairments that significantly affect daily functioning. Upper-limb dysfunction, in particular, contributes markedly to reduced independence and quality of life among stroke survivors [1,2,3].

Stroke risk factors are multifactorial and can be broadly categorized into non-modifiable factors, such as age and sex, and modifiable factors, including cardiovascular and metabolic disorders, obesity, smoking, and alcohol consumption. In addition, several conditions associated with a procoagulant state—such as oncological diseases, post-COVID-19 status, autoimmune disorders, and antiphospholipid syndrome—contribute to stroke occurrence and may be associated with more severe neurological involvement. Beyond their etiological role, these factors are clinically relevant for post-stroke rehabilitation, as they are linked to stroke severity, neuromuscular impairment, and the development of spasticity, which can influence upper-limb motor recovery and responsiveness to rehabilitation interventions [4,5,6].

Moreover, patients with neoplastic pathology, in addition to the increased risk of developing a stroke, also present a degree of cognitive decline, especially if the tumor is located in the brain, an aspect that diminishes active participation in the recovery program [7].

Recent advances in neurorehabilitation have expanded the range of therapeutic strategies available for post-stroke recovery, including robotic-assisted therapy, functional electrical stimulation, and shock wave-based interventions. While each of these modalities has demonstrated potential benefits when applied individually, real-world comparative data evaluating combined rehabilitation protocols—particularly the integration of radial shock wave therapy (RSWT) with functional electrical stimulation (FES) versus conventional electrotherapy—remain limited, especially in patients with upper-limb spasticity [8].

The recovery capacity of the upper limb is much lower than that of the lower limb in terms of time and functional quality [9].

The upper limb offers the capacity for independence, which is why extensive studies are required to develop methods for regaining grip function as early and efficiently as possible. It is known that the upper limb has an average recovery period of 6 months from the onset of the stroke to reach maximum performance in recovering functionality [10].

Early rehabilitation after stroke is grounded in the understanding that stroke-induced brain injury disrupts existing neural activation patterns and synaptic pathways involved in motor control, particularly for the upper limb. Recovery of function depends on the capacity of the central nervous system to reorganize synaptic connections and recruit alternate neural circuits through neuroplastic mechanisms, a process that can be enhanced by task-specific, repetitive, and goal-directed rehabilitation approaches. Disruption of motor pathways often leads to impaired motor memory and coordination, contributing to persistent functional disability beyond the primary lesion. Effective rehabilitation aims to facilitate adaptive neuroplasticity by providing high-intensity, task-oriented practice with sensory feedback, which supports cortical reorganization and improved upper-limb motor outcomes [11,12].

A major cause of disability in the neurological patient with stroke is spasticity, which causes an alteration of active movement. Thus, movement becomes reduced in amplitude and speed of execution. This aspect requires that spasticity be maintained at a low level to allow movements to become fluent and fluid as physiological as possible.

Motor dysfunction also leads to changes in the neurological pattern and in the recovery process; it is necessary for the patient to actively participate in the program to watch and imaginatively contribute to the achievement of the movement through biofeedback techniques [13].

The techniques used in this study used visual feedback and patient participation in the electrostimulation program with the application of robotic kinetic techniques. Effective patient participation is a necessary goal as it leads to the development of neuroplasticity and improved functionality [14].

Through this study, we aim to highlight not only the role of modern techniques but also the fact that the correct application of certain physical therapies, sequentially on target muscle groups of the upper limb, improves functionality and increases grip capacity in post-stroke patients.

## 2. Materials and Methods

### 2.1. Study Design and Ethical Approval

This retrospective observational study analyzed the clinical records of 52 adult patients. The sample size was not pre-determined by an a priori power calculation. Instead, we employed a consecutive sampling method, including all patients who were admitted to the Neuropsychiatry Clinical Hospital of Craiova between February and September 2025 and met our strict inclusion/exclusion criteria. To ensure that this available cohort (*n* = 52) provided sufficient statistical rigor to support our conclusions, we performed a post hoc power analysis using G*Power 3.1.9.7. To minimize selection bias, data was retrieved for all patients who met the inclusion criteria consecutively during the study period. Furthermore, data were extracted from standardized clinical records where assessments had been performed by therapists blinded to the future study objectives. Furthermore, potential confounding bias arising from baseline differences in stroke duration and initial impairment was controlled through the use of analysis of covariance (ANCOVA) in the statistical processing phase.

The study design aimed to evaluate the improvement of prehension using current techniques applied to key muscle groups for upper-limb rehabilitation.

Written informed consent was obtained from each patient. This study was conducted in accordance with the Declaration of Helsinki, and it was approved by the Ethics Committee of Neuropsychiatry Clinical Hospital of Craiova, Romania (number 02/16.01.2025).

### 2.2. Inclusion and Exclusion Criteria

Participants were eligible if they were older than 18 years, developed a stroke confirmed by computer tomography, and had a stroke onset no more than six months prior to enrollment. Patients presenting with neoplasm, cardiovascular, respiratory, or neurological decompensation were excluded as were those with skin lesions contraindicating electrotherapy, individuals with pacemakers or cochlear implants, and patients with recurrent stroke. Both groups received pharmacological treatment tailored to each patient’s needs following the recommendations of neurologists, cardiologists, and other specialists responsible for managing associated conditions.

All patients underwent an initial evaluation and a follow-up assessment at three months. During this interval, they participated in a structured rehabilitation program applying the same assessment scales at both time points. In addition to supervised therapy, all participants were instructed to follow a home-based kinetotherapy program to support functional recovery between sessions.

### 2.3. Clinical Assessment of the Patients and Presentation of Groups

The control group consisted of 26 patients who had received standard care involving the use of galvanic current applied to the upper limb and simple electrostimulation on the flaccid muscles of the upper limb: finger extensors, radial extensor of the carpi longus and brevis, and ulnar extensor of the carpi.

The study group comprised 26 patients whose standardized treatment protocol included galvanic current applications and functional electrostimulation on the flaccid muscles, on the same categories of muscles, in addition to the modern application of shock wave therapy applied to the spastic muscles: deep and superficial flexors of the hand and radial and ulnar flexor of the carpi.

The patients who were included in the study group had a rehabilitation program consisting of galvanic current applications to the paretic upper limb, functional electrical stimulation applied to the flaccid muscles (extensor muscles of upper limb), and shock wave therapy administered to the spastic muscles (flexor muscles of upper limb). In contrast, the patients from the control group had a rehabilitation program consisting of galvanic current applications to the paretic upper limb with classical electrical stimulation for flaccid muscles. The retrospective analysis was structured to allow a comparison between patients treated with modern techniques and those who received classical protocols targeting upper-limb recovery and prehension in stroke patients and to assess the relative effectiveness of each therapeutic method; both methods were usually applied for this pathology in our clinic. Both groups participated in individualized kinetotherapy programs focused on upper-limb functional recovery.

Regarding the environment of origin, the study group included 14 patients (53.8%) from urban areas, while the control group included 13 patients (50%) from similar settings. In terms of age, the study group ranged from 28 to 72 years, with a mean age of 60 years and a median age of 64.5 years (55.3–66.8 years). The control group ranged from 45 to 71 years, with a mean age of 61.5 years and a median age of 63.5 years (56.3–67.8 years).

For the purpose of this study, the primary endpoint was defined as the change in Hand Grip strength at 3 months. Secondary endpoints included changes in spasticity (MAS), muscle force (MRC), and range of motion. Patients admitted to this study were assessed using established evaluation scales targeting multiple aspects of upper-limb function. Muscle strength was evaluated using the Medical Research Council (MRC) scale. Spasticity was assessed with both the Ashworth and Tardieu scales. Prehension ability was measured using a Grip Scale, while finger extension was quantified at the metacarpophalangeal joints. Shoulder mobility was evaluated to determine the range of motion, and pain, including neuropathic pain and pain during shoulder mobilization, was assessed using the Visual Analogue Scale (VAS).

Muscle strength was assessed using the Medical Research Council (MRC) scales from 0 to 5, where 0 means no muscle activity and 5 represents normal strength. The assessment involves segmental muscle strength, a specific assessment for stroke patients [15]. Spasticity is an element that can lead to increased motor deficit precisely due to the decrease in the capacity for controlled active movement. This was evaluated using two complementary scales, the Ashworth scale and the Tardieu scale. The Ashworth scale used in the evaluation considers score 1 without spasticity and score 4 with spasticity with ankylosis [16]. The Tardieu scale adds value to the assessment by quantifying clonus and its persistence [17].

Prehension was assessed using the hand grip test with values from 0 to 3. Quantification of the ability to perform the previous prehension was performed using dynamometer-type devices for each type of movement [16]. The assessment dynamometer values were between 0 and 10.

The extension evaluation was quantified by a goniometer at the metacarpophalangeal joint level and stratified into 3 degrees: from 0 to 35, from 30 to 70 and over 70 degrees of extension using a goniometer [18].

Shoulder mobility represents the range of motion of the shoulder joint. It is well established that stroke patients experience a decrease in shoulder function due to pain during mobilization and the progressive onset of spasticity. Consequently, the range of motion becomes limited, making the preservation of mobility a key objective in upper-limb rehabilitation and in improving overall quality of life [19]. To ensure an accurate quantification of shoulder range of motion, a goniometer was used [20,21,22].

For the assessment of upper-limb pain during shoulder mobilization as well as neuropathic pain, the Visual Analogue Scale (VAS) was used [23].

### 2.4. Work Protocol

All patients received a standard, classic rehabilitation program, with galvanic current electrotherapy (with neurotrophic and analgesic effect), massage, and robotic physiotherapy using a robotic glove.

The ethical criteria regarding the application of modern rehabilitation techniques were respected; all patients received personalized rehabilitation treatment, and no patient was left without rehabilitation treatment.

Galvanization represents a continuous current that, when applied longitudinally to the upper limb, has a neurotrophic and anti-pain effect [24].

Shock wave therapy is a relatively new form of treatment in medical rehabilitation practice for the treatment of chronic pain of the musculoskeletal system, and for the parameters used in this study, it has the effect of reducing spasticity.

The principle of this therapy is to form a ballistic pressure wave (shock wave) through an accelerated compressed air projectile. This is generated by an electronically controlled compressor. The applicator probe transmits kinetic energy to the application area, which is muscle in the case of our study.

**Figure 1:**
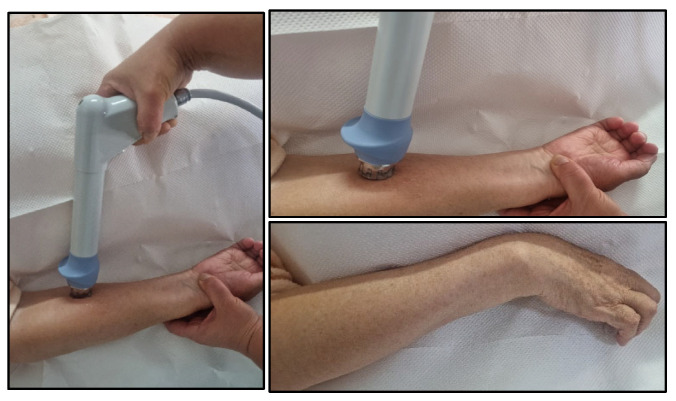
Shock wave application.

There are two main types of shock waves used in therapeutic applications: focused extracorporeal shock waves (ESWT) and radial shock waves (RSWT). The difference between these consists of wave form, propagation speed and shock wave pressure; pulse duration; speed and penetration depth; and concentrated distribution of the shock wave. ESWT is used for precise treatment at the centering point, and RSWT are shock waves applied with controlled pressure on larger areas of action in order to locally model certain rigid, spastic structures, such as in the case of exostoses and the spasticity in our study (Figure 1).

The contraindications of these shock wave applications were taken into account, namely that the patient should not be undergoing anticoagulant treatment and should not have ecchymoses, other local lesions, capillary fragility, coagulation disorders, liver diseases, or changes in the complete blood count with a reduction in the number of platelets.

The applicability of this therapy is diverse from the analgesic and decontracting effect for post-scoliosis muscle contracture or other causes even applied in spasticity [25,26]. The shock waves used were applied in progressive doses: the pressure was increased from 0.5 bar every second session taking into account the patient’s tolerance level and to avoid bruising at the application site, increasing to a maximum of 2 bars. The application area was in the spastic muscle areas, according to the evaluation [16].

We used RSWT for 10 sessions, using the same parameters for all patients. The therapy was initiated with a pressure starting from 0.5 bar and a frequency of 5 Hz longitudinally, gradually increasing from 300 shocks/session to 1000 shocks/session, with a pressure of 1.5 bar and a frequency of 10 Hz at the level of muscle groups.

Following the assessments, we found superior results in favor of the application of RSWT therapy, with increased tolerability of the treatment and no side-effects. Shock wave RSWT therapy is an important tool in the management of spasticity.

Electrostimulation was applied to the extensor muscles of the hand, finger extensors, radial extensor of the carpi longus and brevis, and ulnar extensor of the carpi, preceded and followed by galvanization in the control group. The patient was instructed to watch the movement and to participate actively or imaginatively in the movement.

The control group received simple electrostimulation preceded and followed by galvanization for 10 min.

The study group received galvanization and functional electrostimulation on the same muscle groups, the benefit being that, with each stimulation, the resistance is adapted to the patient’s ability to perform the movement.

Both study groups benefited from modern, high-performance physiotherapy interventions, including the use of a robotic glove for restoring correct motor patterns such as finger prehension and extension, mirror therapy to stimulate kinesthetic image recovery, neuromotor facilitation techniques, and the Bobath concept for spasticity management. Thus, using these kinetic techniques and electrostimulation, both types of movements were controlled: flexion and extension of the fingers and hand with the aim of improving prehension.

### 2.5. Statistical Analysis

Data were saved to an Excel file and analyzed using R packages version 4.3.1. Qualitative data were described using number and percentages. Quantitative data were described using mean ± standard deviation, median (interquartile range, IQR) and range. The McNemar test was performed to compare paired samples in terms of a dichotomous variable. To compare paired means for continuous data that are not normally distributed, we used the non-parametric Wilcoxon signed-ranks test, whereas for normally distributed data, we used the paired samples *t*-test. The Shapiro–Wilk test was used to verify the normality of the continuous data. Quantitative variables were handled based on their distribution and scale type. For clinical outcomes measured on ordinal scales (e.g., MRC, Hand Grip, and MAS), non-parametric methods were prioritized. In our comparative analysis, baseline clinical scores and time since stroke onset were utilized as continuous covariates in analysis of covariance (ANCOVA) models to provide adjusted estimates and minimize the impact of initial group imbalances.

To account for multiple statistical testing across the various functional scales, a Bonferroni correction was applied, and the adjusted *p*-values are reported where applicable. To address baseline imbalances in stroke chronicity and initial impairment severity, one-way ANCOVA was employed for primary outcomes (Hand Grip and Dynamometry) and spasticity (MAS). Group (Study vs. Control) served as the fixed factor, while time since stroke onset, baseline Fugl–Meyer scores, and baseline values of the respective outcome were included as covariates. This allowed for the calculation of estimated marginal means and 95% confidence intervals (CI). Effect sizes were quantified using Cohen’s *d* (derived from adjusted means), where Cohen’s *d* of 0.2, 0.5, and 0.8 represent small, medium, and large effects, respectively. This approach was chosen to minimize the risk of regression to the mean and to adjust for the differential recovery trajectories often associated with varying levels of initial impairment.

The statistical significance of the obtained results was set at 0.05. To minimize the risk of Type I errors arising from multiple comparisons between clinical scales (e.g., MRC, MAS, Hand Grip, and VAS), the Bonferroni correction was applied. Adjusted *p*-values (*p_adj_*) were calculated by multiplying the original *p*-value by the number of comparisons made within each clinical domain, and these adjusted values are reported where applicable.

A post hoc power analysis was conducted using G*Power (version 3.1.9.7) to evaluate the adequacy of the sample size (*n* = 52).

## 3. Results

The demographic and clinical data of patients who participated in this study is shown in Table 1. Both groups showed no significant difference regarding age, gender, environment and HandEx (*p*-value > 0.05).

Baseline characteristics were compared to ensure the groups were sufficiently homogeneous to evaluate the differences in therapeutic outcomes. Statistical analysis revealed that the two groups were comparable regarding age (61.5 ± 7.11 years for the study group vs. 60 ± 11.7 years for the control group; *p* = 0.963), gender distribution (50% male in both), and urban environment (*p* = 0.783). However, the study group presented with a significantly higher mean time since stroke onset compared to the control group (3.27 ± 1.43 months vs. 2.08 ± 0.98 months; *p* = 0.002). Significant baseline differences were also observed in clinical severity: the study group exhibited higher levels of spasticity on the Modified Ashworth Scale (*p* = 0.013) and lower functional performance, with significantly lower scores in Hand Grip (0 vs. 0.28 ± 0.28; *p* < 0.001) and Dynamometry (0.12 ± 0.15 vs. 0.39 ± 0.54; *p* = 0.008). These differences indicate that the study group initially presented with more severe motor impairment and a more chronic phase of recovery than the control group.

Hand Grip was significantly increased after treatment with shock wave and FES, as shown in Table 2 (from 0.28 ± 0.28 to 0.99 ± 0.36, *p*-value < 0.001). Also, Hand Grip was significantly increased in the study group (from 0 to 0.46 ± 0.48, *p*-value = 0.001), as shown in Table 3. There was a significant increase in Hand Grip after the shock wave and FES therapy compared to the patients without these therapies: 0.71 (95%CI, 0.57–0.85) vs. 0.46 (95%CI, 0.27–0.66). Even when we mathematically account for the fact that the study group started with more severe spasticity and a longer time since stroke, the FES + RSWT treatment still produced better results, suggesting that the treatment effect persists even when initial clinical advantages in the control group are accounted for. A moderate effect size was observed for Hand Grip (Cohen’s *d* = 0.335, 95%CI [−0.421, 1.09]), and a large effect size was observed for dynamometry (Cohen’s *d* = 1.85, 95%CI [1.00, 2.70]). The ANCOVA proves that the “superiority” of the study group is a true treatment effect rather than a result of starting with worse scores (floor effect).

**Figure 2:**
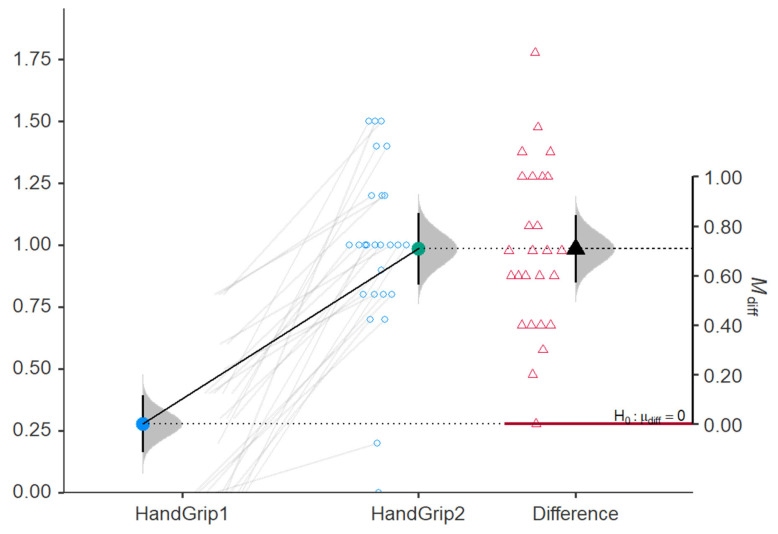
Comparison of Hand Grip before (HandGrip1) vs. at 3 months after (HandGrip2) shock wave therapy. Red is the color for null hypothesis (no differences between HandGrip1 and HandGrip2). Blue circle is the mean of HandGrip1 (the black line represents 95%CI of HandGrip1). Green circle is the mean of HandGrip2 (the black line represents 95%CI of HandGrip2). Black triangle is the mean of the differences between HandGrip1 and HandGrip2 (the black line represents 95%CI of the differences). Grey shaded areas are the distributions used to calculate confidence intervals. The red line marks zero-reference. Dashed Line is the comparison line that bridges the gap between the two vertical axes, showing how the “After” group relates to the “Difference” scale.

These differences between the two groups are due to the application of shock wave therapy and functional electrostimulation, which further improved the prehension capacity quantified by Hand Grip, by modifying the quality of the muscle, increasing elasticity and reducing the degree of spasticity (Figure 2).

The results obtained show the reduction in spasticity quantified by two scales, Asworth and Tardieu, with significant results in the study group, where there was also a significant improvement in the degree of prehension measured by using the Hand Grip scale.

**Figure 3:**
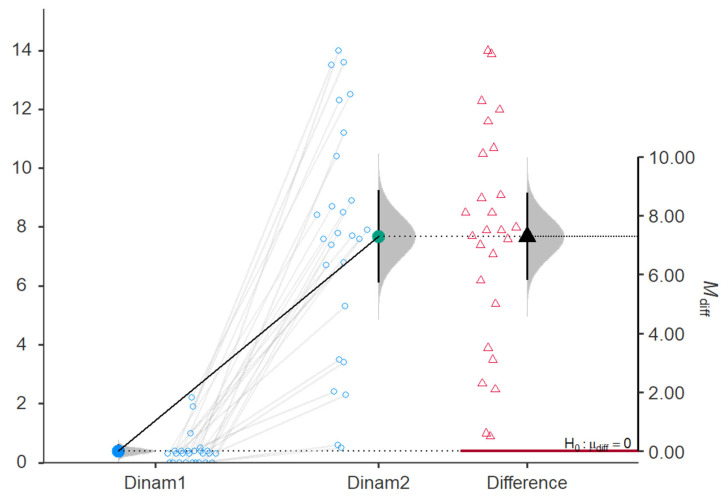
Comparison of Dinam before (Dinam1) vs. at 3 months after (Dinam2) shock wave therapy and FES.

**Figure 4:**
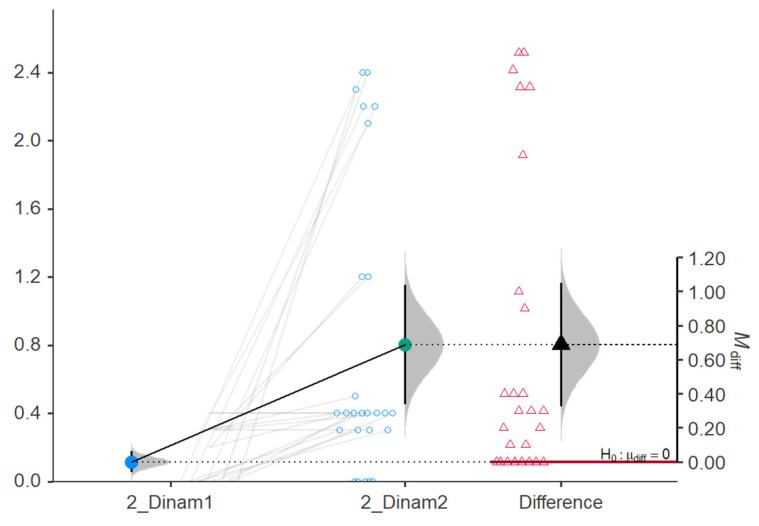
Comparison of Dinam in the group of patients without shock wave and FES therapy (baseline vs. at 3 months).

Dinam was significantly increased after treatment with shock wave and FES, as shown in Table 2 (from 0.39 ± 0.54 to 7.67 ± 3.89, *p*-value < 0.001). Also, Dinam was significantly increased in the study group (from 0.12 ± 0.15 to 0.8 ± 0.87, *p*-value < 0.001), as shown in Table 3. There was a significant increase in Dinam after the shock wave therapy and FES compared to the patients from the control group: 7.28 (95%CI, 5.8–8.77) vs. 0.69 (95%CI, 0.33–1.05), as shown in Figure 3 and Figure 4. To account for baseline differences in stroke duration and initial impairment, an ANCOVA was performed. For the primary outcome of Hand Grip strength, the adjusted post-treatment mean for the study group was significantly higher than the control group.

Muscle strength in the dynamometer assessment improved in the study group by increasing the muscle’s ability to increase its length once spasticity was reduced by applying serial shock wave sessions and stimulated by applying FES.

In the study group, the improvement was noticed at follow-up for all marked indicators, as shown in Table 2. Using G*Power post hoc power analysis for Hand Grip, the calculated effect size (Cohen’s *d*) was 4.36, yielding a statistical power of 0.99. This indicates that the study had a 99% probability of detecting a true effect, satisfying the standard requirement for clinical research power (>0.80).

The study group that underwent shock wave treatment obtained much better results compared to the control group, both regarding spasticity and functionality quantified by prehension and the degree of extension of the hand using the goniometer.

In the group of patients treated without shock wave therapy and FES, the improvement was noticed at follow-up for all marked indicators, without Tardieu, as shown in Table 3.

The medical ethics conditions were respected by which both groups received adequate physiotherapy treatment, the homogeneity of the groups, the onset of a maximum of 6 months from the stroke at the first evaluation and the maintenance of the same therapy conditions at the 3-month follow-up. Thus, we can note and highlight the role of combined therapy and the use of modern techniques as an added value with obvious and quantifiable results. The control group, through the applied treatment, obtained notable results, but the results were inferior to the study group, which had statistically significantly better results in terms of spasticity and functional capacity assessed by Hand Grip. After adjusting for baseline severity imbalances using an analysis of covariance (ANCOVA) model, the study group still maintained significantly higher gains in Hand Grip performance (*p* < 0.01).

Shoulder mobility was assessed using a goniometer, with flexion, rotation, and abduction considered the most relevant movements for performing activities of daily living (ADL). Rotation was quantified by summing internal rotation, measured at 90°, with external rotation, also measured at 90°, resulting in a total rotational range of 180°.

The results indicated that the study group experienced a slight improvement in shoulder mobility, showing only a small difference compared to the control group. These findings suggest that shoulder functionality improved as a result of the applied kinetotherapy programs.

**Figure 5:**
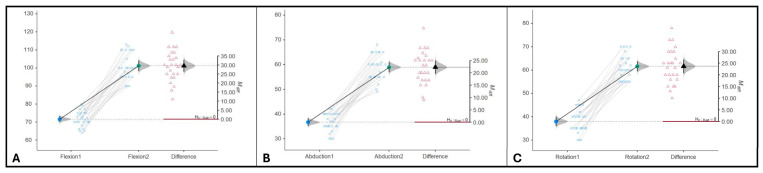
Control group differences. (**A**). Flexion. (**B**). Abduction. (**C**). Rotation. The filled black triangle shows the point estimate of the mean difference. The horizontal red line represents the null hypothesis. The vertical error bar around the mean difference represents the 95%CI.

The observed differences indicated highly statistically significant improvement (*p* < 0.001) of flexion (*M* _diff_ = 29.5, 95%CI: [26.2, 32.9], as shown in Figure 5A), abduction (*M* _diff_ = 22.2, 95%CI: [19.6, 24.9], as shown in Figure 5B) and rotation (*diff*/*M* _diff_ = 23.6, 95%CI: [20.6, 26.6], as shown in Figure 5C) in the control group.

**Figure 6:**
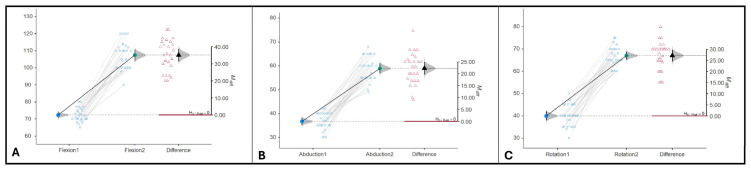
Study group differences. (**A**). Flexion. (**B**). Abduction. (**C**). Rotation.

The observed differences indicated highly statistically significant improvement (*p* < 0.001) of flexion (*M* _diff_ = 35.1, 95%CI: [31.4, 38.8], as shown in Figure 6A), abduction (*M* _diff_ = 29.2, 95%CI: [27.0, 31.3], as shown in Figure 6B) and rotation (*M* _diff_ = 27.1, 95%CI: [24.5, 29.7], as shown in Figure 6C) in the study group.

Circumduction, which best reflects shoulder functionality, demonstrated near-normal improvement in the study participants. Assessment of shoulder pain revealed significantly higher improvements in the study group compared to the control group. This suggests that the enhancement of hand functionality through modern therapeutic interventions may indirectly influence the patient, leading to a reduced perception of pain.

The results showed a 64.07% improvement in the study group, whereas the control group exhibited a 27.7% rate of improvement.

**Figure 7:**
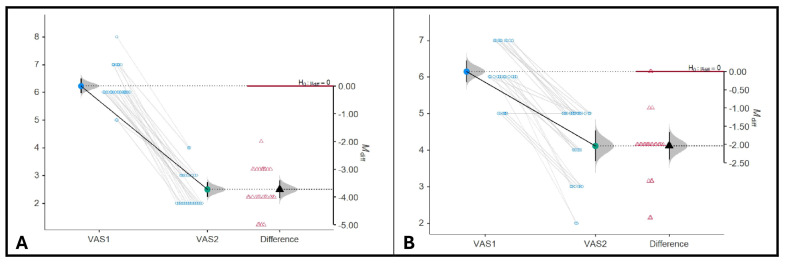
Visual Analogue Scale (VAS) assessment. (**A**). Study group. (**B**). Control group.

Both groups showed improvements in upper-limb function and shoulder mobility after the rehabilitation programs. However, the study group (Figure 7A), which received galvanic current, functional electrical stimulation (FES) to flaccid extensors, and shock wave therapy to spastic flexors, demonstrated significantly greater gains in active shoulder range of motion (ROM) compared to the control group (Figure 7B).

Regarding pain assessment using the VAS, patients in the study group reported a more pronounced reduction in both neuropathic pain and pain during shoulder mobilization (*M* _diff_ = −3.73, 95%CI: [−4.05, −3.42], *p* < 0.001). In contrast, the control group (Figure 7B), which received galvanic current and classical electrical stimulation, showed moderate improvement in shoulder mobility and pain reduction (*M* _diff_ = −2.04, 95%CI: [−2.41, −1.67], *p* < 0.001), but the changes were less marked than in the study group.

## 4. Discussion

The first medical uses of ESWT were for kidney stones starting in 1980 [27]. Currently, the ISMST (International Society for Medical Shockwave Treatment) has approved the following standard applications of the therapy in musculoskeletal pathology. In the last decade, due to the physiological properties that emerged following the application of the therapy (regeneration of degenerated tissue, neovascularization, formation of free radicals, change in the permeability of cell membranes, formation of nitric oxide and variable increase in growth factor), it was decided to broaden the spectrum of clinical application of ESWT, one of which is spasticity [28].

Mariotto et al. believe that ESWT can induce the synthesis of nitric oxide, involved in the formation of the neuromuscular junction in the peripheral nervous system and plays important roles in neurotransmission and synaptic plasticity in the central nervous system [29]. Although several experimental and clinical studies have suggested that extracorporeal shock wave therapy may influence neuromuscular function through mechanisms involving nitric oxide signaling, angiogenesis, and modulation of neuromuscular junction activity, these pathways were not directly assessed in the present study. Current evidence indicates that nitric oxide may play a role in muscle tone regulation and peripheral tissue remodeling following shock wave application; however, such effects have been primarily demonstrated in preclinical models or indirect clinical observations. Therefore, nitric oxide-related mechanisms should be regarded as hypothesized explanatory pathways supported by previous literature rather than empirically demonstrated contributors to the functional improvements observed in our cohort [1,2,3]. Further studies incorporating biochemical or imaging biomarkers are required to directly elucidate the role of nitric oxide in post-stroke spasticity modulation [8,30].

Another interesting study published in March 2012, performed on rats, shows the importance of reducing spasticity by inhibiting nerve transmission at the neuromuscular junction after applying ESWT, thus demonstrating the basic role that nitric oxides seem to play in the mechanisms of spasticity improvement [31].

Following the positive therapeutic effects of ESWT on bone and tendon, Manganotti and Amelio considered that the reduction in spasticity is due to the direct action on muscle fibrosis produced by chronic hypertonia [32]. Thus, Manganotti and Amelio and Yoo et al. reported that the therapeutic effect of ESWT on upper-limb spasticity could last at least 4 weeks and a maximum of 12 weeks, while other authors observed only an immediate effect, with spasticity returning after 3 days. The reason for the differences in the results of these studies mentioned seems to be the mechanism of shock wave generation, the energy per unit area, the number of applications, the place of application and the period since the patient complained of dysfunction. Our study demonstrates the usefulness of applying EWST combined with the rest of the rehabilitation program. Despite these limitations, ESWT may be a useful alternative in the treatment of spasticity, as it is a non-invasive procedure with fewer adverse effects compared to existing treatment methods [33].

Because both the modern and classical protocols were part of the standard rehabilitation options available at our clinic, this retrospective analysis allowed us to evaluate real-world efficacy without interfering with clinical decision-making. The described study presents a comprehensive investigation of prehension rehabilitation in stroke patients, intending to develop accurate and applicable therapeutic methods for upper-limb recovery. It included 52 study patients divided into two equal groups (study group and control group) of 26 patients. Both groups received galvanic electrotherapy, therapeutic massage, and robotic kinetotherapy. The study group received functional electrostimulation on flaccid upper-limb muscles and shock waves on spastic muscles in progressive dosages compared to the control group.

Prehension rehabilitation plays a major part in motor recovery for upper limbs after stroke. Conventional stroke rehabilitation primarily includes physical therapy, functional electrical stimulation on flaccid muscles, shock wave therapy on spastic muscles and robotic kinetotherapy along with drug therapies [34]. However, residual functional disabilities still affect many stroke survivors despite all these therapies as they continue to have difficulties in performing daily activities [35]. It is known that the strength of a muscle is greater the longer its length and the larger its cross-sectional area are, and in the case of our study, only the length could be influenced by reducing spasticity. Various technology-based stroke rehabilitation interventions, including prehension rehabilitation, have been developed in the last few decades, showing promising results in improving stroke patients’ hand grip, functional mobility and independence [24].

Our results demonstrated significant statistical improvement in hand grasp in patients who received functional electrostimulation on flaccid upper-limb muscles and shock waves on spastic muscles compared to patients who received only therapeutic massage and galvanic electrotherapy. Similar results were obtained by Guo J et al. on a large study group of more than 100 patients using shock wave therapy in the recovery of upper-limb spasticity for stroke patients [36]. Moreover, Wang CJ et al. observed that a single acoustic wave impulse stimulated the healing and regeneration of soft tissue by inhibiting pain receptors’ function and increasing blood flow by stimulating angiogenesis. Therefore, shock wave therapy produced an improvement in musculoskeletal healing [37].

Spasticity has a negative effect on post-stroke patients‘ quality of life, as it interferes with their ability to be independent, and can lead to the development of depressive symptoms [38]. Although improvements in upper-limb function, spasticity reduction, and pain relief may indirectly influence patients’ daily functioning, no patient-reported outcome measures or validated quality-of-life questionnaires were included in the present study. Consequently, the impact of the observed functional improvements on quality of life cannot be directly assessed or inferred from the available data. Future studies should incorporate standardized patient-reported measures to better evaluate the relationship between functional recovery and quality-of-life outcomes in post-stroke rehabilitation.

Extracorporeal shock wave therapy is gaining attention as a promising, non-invasive adjunct in post-stroke rehabilitation, particularly for managing spasticity and improving motor function. A 2019 meta-analysis of eight randomized controlled trials (301 patients) found that ESWT significantly reduced muscle tone (Modified Ashworth Scale: WMD = −0.36), decreased pain (VAS: WMD = −0.94), and enhanced both range of motion (+5.97°) and motor control measured by Fugl–Meyer (WMD = +1.26) [8,39]. Systematic reviews consistently report that both focused and radial extracorporeal shock wave therapy lower spasticity, boost muscular and postural function, and increase neuromuscular endurance. Extracorporeal shock wave therapy influences neuroplasticity, modulates inflammatory cytokines, stimulates angiogenesis by releasing growth factors and also acts on the neuromuscular junction by temporarily decreasing motor neuron excitability. Although protocols vary, preliminary randomized trials suggest optimal results occur when shock waves are applied both at muscle–tendon junctions and intramuscularly, resulting in measurable gait improvements [40,41,42].

Manganotti et al. [40] demonstrated that the beneficial effects of shock wave therapy in stroke patients can last up to 12 weeks after treatment, while other authors noticed that the spasticity was reduced up to 4 weeks [43,44]. Shock wave therapy interventions for spastic muscles demonstrated positive effects when applied as early as 1 week after stroke [45]. Extracorporeal shock wave therapy represents a low-risk and encouraging therapeutic modality that supports classical therapies and promotes recovery after stroke [46]. Shock wave therapy represents a promising, non-invasive treatment for stroke rehabilitation, offering new hope to patients with limited recovery as it is based on acoustic waves that stimulate healing in targeted tissues and consequently improves the motor function by reducing spasticity and stimulating neuroplasticity in post-stroke patients [47].

Electrostimulation represents a potential effective treatment in recovering movement control and functional ability after stroke [48,49]. Lutokin et al. conducted a study on a similar number of stroke patients compared to our study, divided into two groups (a study group that received exoskeleton with functional electrostimulation and a control group that received only exercise therapy). Their results demonstrated that the study group patients had a significant improvement in movement control compared to the control group [50]. Moreover, Ambrosini et al. conducted a study on 72 patients, showing that functional electrical stimulation combined with hybrid robotic systems positively influenced upper-limb motor recovery after stroke [51]. Functional electrical stimulation combined with robot-assisted upper-limb training proved to be more effective on neuromuscular rehabilitation compared to robot-assisted upper-limb training alone, as shown by Xu yang et al. on a group of 60 patients with stroke enrolled in the study between one week and less than a year after stroke onset [52].

Current research shows that robotic-assisted arm training alone did not demonstrate superior results compared to conventional therapy in terms of upper-limb spasticity. Indirect comparisons suggest that there is no robotic device superior to other devices for hand–arm recovery [53].

Stroke comes along with multiple side-effects, and the musculoskeletal ones have a major impact on patients’ quality of life. Spasticity arises due to an imbalance between the inhibitory and excitatory influences of descending pathways on the stretch reflex. Unfortunately, the genetic contribution to the development of spasticity following a stroke remains poorly understood at this time [25,54].

In our study, we observed an improvement in shoulder mobility in the study group compared to the control group, suggesting that modern, targeted rehabilitation interventions can positively influence functional recovery of the hemiparetic shoulder. We must acknowledge that the study group initially exhibited a more severe clinical profile, characterized by lower baseline Hand Grip scores (floor effect) and a longer duration since stroke onset compared to the control group. In a non-randomized retrospective setting, such imbalances could potentially inflate the perceived efficacy through regression to the mean. However, our adjusted analysis (ANCOVA) confirms that the magnitude of improvement in the study group—moving from a non-functional state to measurable prehension—remains statistically significant. While this association suggests a benefit of the RSWT + FES protocol, the retrospective nature of this work means that unmeasured confounding factors may still influence these results, and our findings should be interpreted as a strong clinical association rather than absolute causal superiority. These findings align with previous evidence indicating that tailored mobilization and therapy can preserve or restore range of motion (ROM) in stroke survivors. For instance, a randomized trial demonstrated that a combined soft-tissue mobilization technique was effective in increasing passive external rotation of the shoulder in subacute stroke patients [55]. Pain remains a critical barrier to rehabilitation in stroke patients. Hemiplegic shoulder pain (HSP) is highly prevalent and associated with poorer functional outcomes [56]. The combination of functional electrical stimulation and shock wave therapy may have contributed to pain reduction by modulating muscle tone and reducing spasticity, indirectly facilitating more comfortable mobilization. This is supported by prior work where robotic-assisted therapy significantly reduced shoulder pain and improved passive ROM compared to conventional physiotherapy [57].

Recent research has shown that post-stroke hypertonia exhibits position-dependent resistance to passive displacement, suggesting that spasticity is not a uniform phenomenon but varies across the workspace of the limb [58]. Our therapeutic approach, which includes shock wave therapy for spastic muscles, may exert its benefits in part by altering neuromuscular excitability or local soft-tissue properties, thereby improving passive and possibly active mobility over time.

The limitations of our current study arise from the variability in how spasticity is assessed in individual patients. Spasticity is a dynamic syndrome, which can be negatively influenced by multiple factors such as low temperature, emotions, and stress. Other limitations of the current study relate to the fact that electrostimulation programs and shock wave therapy parameters vary depending on the patient’s response to stimulation and spasticity presentation. Finally, another limitation of the current study is the relatively small number of patients that formed the study and control groups.

Our study introduces a novel approach by combining FES and RSWT for improving upper-limb motor functionality in stroke patients in comparison to classical rehabilitation methods, such as robotic kinesiotherapy with a robotic glove and therapeutic massage. Although the combined application of radial shock wave therapy and functional electrical stimulation was associated with greater functional improvements in the present cohort, no formal statistical interaction analysis was performed to evaluate synergistic effects between the two interventions. Therefore, the observed benefits should be interpreted as reflecting potential complementary effects of targeting spastic flexor muscles with RSWT and facilitating activation of weakened extensor muscles through FES rather than true synergy. Future prospective studies with factorial designs and predefined interaction analyses are required to formally assess synergistic effects between these rehabilitation modalities. RSWT likely reduces the rheological component of spasticity in the flexor muscles by disrupting collagen cross-links and inducing nitric oxide synthesis. By lowering this ‘peripheral resistance,’ the subsequent application of FES to the antagonist extensor muscles becomes more effective, facilitating neuroplasticity and the reorganization of motor maps in the motor cortex.

Although some benefits were also registered for the control group, the added functional electrostimulation and shock wave therapy had superior results in improving functional capacity of the upper limb. The current study was conducted on homogeneous groups of equal size. In summary, our findings support the concept that a multimodal rehabilitation program—encompassing functional electrical stimulation, shock wave therapy, and individualized mobilization exercises—has functional gains in shoulder mobility after stroke. These improvements may, nevertheless, contribute to enhanced prehension and overall upper-limb function, potentially translating into better quality of life.

## 5. Conclusions

In our retrospective analysis, patients who underwent a rehabilitation program combining radial shock wave therapy and functional electrical stimulation demonstrated greater improvements in upper-limb motor function, hand grip strength, and spasticity-related outcomes compared with those receiving conventional electrotherapy alone. These findings suggest that targeting spastic flexor muscles with RSWT alongside activation of weakened extensor muscles through FES may offer complementary functional benefits in post-stroke upper-limb rehabilitation. Future prospective studies are necessary in order to confirm these findings and to better define the role of combined RSWT and FES within comprehensive post-stroke rehabilitation strategies.

## Figures and Tables

**Table 1:** Demographic and clinical data of the studied groups and characteristics of the patients.

Baseline Characteristics	Total Cohort (*n* = 52)	Control Group (*n* = 26)	Study Group (*n* = 26)	*p*-Value
Age, years				0.963
Mean ± SD	60.7 ± 9.62	60 ± 11.7	61.5 ± 7.11	
Median (IQR)	64 (56–67)	64.5 (55.3–66.8)	63.5 (56.3–67.8)	
Range	28–72	28–72	45–71	
Gender, male	26 (50%)	13 (50%)	13 (50%)	1.0
Environment, urban	27 (51.9%)	14 (53.8%)	13 (50%)	0.783
Onset, months				0.002
Mean ± SD	2.67 ± 1.35	2.08 ± 0.98	3.27 ± 1.43	
Median (IQR)	3 (2–3.25)	2 (1–3)	3 (2–4)	
Range	1–6	1–4	1–6	
FMS				0.015
1	26 (50%)	9 (34.6%)	17 (65.4%)	
2	23 (44.2%)	14 (53.8%)	9 (34.6%)	
3	3 (5.8%)	3 (11.5%)	0	
Modified Ashworth Scale				0.013
1	7 (13.5%)	4 (15.4%)	3 (11.5%)	
2	29 (55.8%)	19 (73.1%)	10 (38.5%)	
3	16 (30.8%)	3 (11.5%)	13 (50%)	
Tardieu				0.014
1	3 (5.8%)	1 (3.8%)		
2	33 (63.5%)	22 (84.6%)		
3	16 (30.8%)	3 (11.5%)		
HandEx				0.392
0	33 (63.5%)	15 (57.7%)	18 (69.2%)	
1	19 (36.5%)	11 (42.3%)	8 (30.8%)	
Hand Grip	0.14 ± 0.24	0.28 ± 0.28	0	<0.001
Dinam	0.25 ± 0.42	0.39 ± 0.54	0.12 ± 0.15	0.008

**Table 2:** Scales measured in the study group.

Study Group (with Shock Wave Therapy)	Baseline, Before Shock Wave	At 3 Months After Shock Wave Therapy	At 3 Months (adj. Mean ± SE)	Effect Size (Cohen’s *d*, 95%CI)	*p*-Value
Hand Grip	0.28 ± 0.28	0.99 ± 0.36	0.78 ± 0.08	0.335 (−0.42, 1.09)	<0.001
Dinam	0.39 ± 0.54	7.67 ± 3.89	6.47 ± 0.56	1.85 (1.0–2.7)	<0.001
FMS					<0.001
1	9 (34.6%)	0			
2	14 (53.8%)	2 (7.7%)			
3	3 (11.5%)	16 (61.5%)			
4	0	8 (30.8%)			
Ashworth					<0.001
1	4 (15.4%)	14 (53.8%)			
2	19 (73.1%)	12 (46.2%)			
3	3 (11.5%)	0			
Tardieu					<0.001
1	1 (3.8%)	14 (53.8%)			
2	22 (84.6%)	12 (46.2%)			
3	3 (11.5%)	0			
HandEx					<0.001
0	15 (57.7%)	0			
1	11 (42.3%)	5 (19.2%)			
2	0	14 (53.8%)			
3	0	7 (26.9%)			

**Table 3:** Scales measured in the group of patients treated without shock wave therapy.

Control Group (Without Shock Wave Therapy)	Baseline	At 3 Months	At 3 Months (adj. Mean ± SE)	Effect Size (Cohen’s *d*, 95%CI)	*p*-Value
Hand Grip	0	0.46 ± 0.48	0.66 ± 0.08	0.335 (−0.42, 1.09)	0.001
Dinam	0.12 ± 0.15	0.8 ± 0.87	2.01 ± 0.56	1.85 (1.0–2.7)	<0.001
FMS					<0.001
1	17 (65.4%)	5 (19.2%)			
2	9 (34.6%)	13 (50%)			
3	0	8 (30.8%)			
4	0	0			
Ashworth					0.006
1	3 (11.5%)	4 (15.4%)			
2	10 (38.5%)	16 (61.5%)			
3	13 (50%)	6 (23.1%)			
Tardieu					0.073
1	2 (7.7%)	3 (11.5%)			
2	11 (42.3%)	14 (53.8%)			
3	13 (50%)	9 (34.6%)			
HandEx					0.02
0	18 (69.2%)	12 (46.2%)			
1	8 (30.8%)	14 (53.8%)			

## Data Availability

The original contributions presented in this study are included in the article. Further inquiries can be directed to the corresponding author.

## Abbreviations

The following abbreviations are used in this manuscript:

FES	Functional Electrical Stimulation
WHO	World Health Organization
ESWT	Extracorporeal Shock Wave
RSWT	Radial Shock Wave
MRC	Medical Research Council
ADL	Daily Life Activity
VAS	Visual Analogue Scale
IQR	Interquartile Range
ISMST	International Society for Medical Shockwave Treatment
